# Author Correction: Linking drug target and pathway activation for effective therapy using multi-task learning

**DOI:** 10.1038/s41598-019-43503-0

**Published:** 2019-05-03

**Authors:** Mi Yang, Jaak Simm, Chi Chung Lam, Pooya Zakeri, Gerard J. P. van Westen, Yves Moreau, Julio Saez-Rodriguez

**Affiliations:** 1 RWTH Aachen University, Faculty of Medicine, Joint Research Center for Computational Biomedicine, Aachen, Germany; 2 European Molecular Biology Laboratory, European Bioinformatics Institute, Wellcome Trust Genome Campus, Cambridge, CB10 1SA UK; 3 ESAT-STADIUS, KU Leuven B-3001, Heverlee, Belgium; 4 Division of Drug Discovery and Safety, Leiden Academic Centre for Drug Research, Leiden University, Einsteinweg 55, 2333CC Leiden, The Netherlands

Correction to: *Scientific Reports* 10.1038/s41598-018-25947-y, published online 29 May 2018.

In the Supplementary Information file originally published with this Article, Supplementary Figures S1 to S9 were omitted. These errors have been corrected in the Supplementary Information that now accompanies the Article.

