# Supplementary material for: Linking drug target and pathway activation for effective therapy using multi-task learning
Source: Sci Rep. 2018 May 29;8:8322. doi: 10.1038/s41598-018-25947-y (PMC5974390; doi:10.1038/s41598-018-25947-y)
Supplement: Supplementary file 1 — Supplementary Information [file 41598_2018_25947_MOESM1_ESM.pdf]

Supplementary material to

Linking drug target and pathway activation for effective therapy using multi-task learning

Mi Yang[1], Jaak Simm[3], Chi Chung Lam[4], Pooya Zakeri[3], Gerard J.P. van Westen[4], Yves Moreau[3], Julio Saez-Rodriguez[1,2]

[1]RWTH Aachen University, Faculty of Medicine, Joint Research Center for Computational Biomedicine, Aachen, Germany.

[2]European Molecular Biology Laboratory, European Bioinformatics Institute, Wellcome Trust Genome Campus, Cambridge CB10 1SA, UK.

[3]ESAT-STADIUS, KU Leuven B-3001 Heverlee, Belgium.

[4]Division of Drug Discovery and Safety, Leiden Academic Centre for Drug Research, Leiden University, Einsteinweg 55, 2333CC, Leiden, The Netherlands

Supplementary information

Supplementary analysis 1: Drug response prediction in different settings

While our aim was to use Macau to obtain interpretable results rather than improve predictability, we first compared the performance of Macau to standard linear regression (ridge/lasso) and tree based non linear regression such as Random Forest and XGBOOST (**Supplementary Table S2**). When building models to predict drug response taking into account multiple drugs and cell lines, one can define four differents settings which mirror different use cases (**Supplementary Fig. S1**). As cell line descriptor, we used gene expression, as well as PROGENy[12] pathway scores. PROGENy is a data driven pathway method aiming at summarizing high dimensional transcriptomics data into a small set of pathway activities (**Methods**). The 11 PROGENy pathways currently available are EGFR, NFkB, TGFb, MAPK, p53, TNFa, PI3K, VEGF, Hypoxia, Trail and JAK STAT. For setting 1, 2 and 4, we used 10 fold cross validation, repeated 10 times. We define the prediction performance as the Pearson's Correlation (r) of observed versus predicted drug response (IC50).

Setting 1: Prediction of new cell lines for existing drugs

The meaning of this framework is to start with a subset of drugs and assign them to the right patient, e.g. new patients based on their genomic information (**Supplementary Fig. S1a, Supplementary Table S1**). We tested three different input datasets for the cell lines: (i) complete gene expression, (ii) PROGENy scores, and (iii) a combination of Single Nucleotide Polymorphism (SNP) and Copy Number Variation (CNV) (**Supplementary Fig. S2a**). We define the prediction performance as the Pearson's Correlation (r) of observed versus predicted drug response (IC50). Gene expression performed best, with an average correlation for all 265 drugs of r=0.40 (10 fold cross validation, repeated 10 times), not surprisingly as it uses all 17419 genes. PROGENy has lower performance (r=0.30), specially considering its low dimension (only 11 pathways). SNP/CNV performs the worst (r=0.21) despite a dimension of 735. These results supports the use of gene expression derivative methods as predictive input features, in agreement with previous studies [2,13].

We then compared multitask Macau with standard single task linear regression, Random Forest and XGBOOST. Using gene expression and drug target, there is no significant difference between Macau (r = 0.40) and LASSO (r = 0.41), p = 0.39. For PROGENy scores, there is no significant difference between Macau (r=0.30) and Ridge (r=0.30), p = 0.92. Random Forest and XGBOOST performed the same with r=0.31, comparable to Macau and ridge. Finally for SNP/CNV, Macau performed significantly better than Ridge (p = 0.00051).

Setting 2: Prediction of new drugs on existing cell lines

A second important scenario is to predict the effect of a new drug on a set of patients based on the side information of the drug. If the new drug is predicted to be better than the existing ones, then a therapeutical switch can be considered. The concept of "new drug" is relative to the patient, it can concern existing drugs which have never been used for a patient group (**Supplementary Fig. S1b, Supplementary Table S1**)

As a benchmark, we compared Macau with standard Ridge regression (**Supplementary Fig. S2b**). To be able to predict the effect of new drugs, we considered as additional side features ECFP4 chemical fingerprints [14]. The average correlation with Macau for the cell lines is 0.42 with drug target and 0.28 with ECFP4, in both cases significantly better than Ridge regression (r=0.12; $p < 2.2e^{-16}$, and r=0.05; $p < 2.2e^{-16}$, respectively). Random Forest and XGBOOST performed better than ridge with r=0.38 and r=0.19, respectively. But Macau still performed significantly better than Random Forest ($p < 2.2e^{-16}$).

Setting 3: Prediction of existing drugs and existing cell lines (**Supplementary Fig. S1c, Supplementary Table S1**)

In this setting we solve an imputation problem, where the test set is randomly chosen from the drug response matrix. We can use side information from both sides to improve the result. We tested setting 3 on GDSC (**Supplementary Table S3**) datasets. In overall, we were able to get an excellent prediction: mean r=0.932 with 90% of the data as training set and 10% and even r=0.834 with 99% as test set.

Setting 4: Prediction of new drugs on new cell lines (**Supplementary Fig. S1d, Supplementary Table S1**)

This setting aims at predicting a new drug's effect on a new cell line solely based on drug target information and whole transcriptomics, hence a very challenging task. We used 2 simultaneous 10-fold cross validation of drugs and cell lines, obtaining an average correlation of r=0.45 (sd= 0.14) for Macau. When replacing transcriptomics with PROGENy scores, Macau performed at r=0.42 (sd=0.15). We obtained similar result using Elastic net and Random Forest with PROGENy (r=0.41, sd=0.12; r=0.42, sd=0.17, respectively). XGBOOST performed lower with r=0.39 (sd=0.17).

Supplementary analysis 2: Additional literature validation for the interaction matrix

Aerodigestive tract

We observe a clear pattern of 3 protein targets (EGFR, ERBB2 and HDAC1) having strong sensitive interaction with activation of EGFR pathway (**Supplementary Fig. S3a**). Targeting EGFR/ERBB1 confers sensitivity when EGFR pathway is activated is no surprise.

We also found sensitive interaction between VEGF pathway and protein HDAC1 (**Supplementary Fig. S3a**). Synergy between VEGF and anti HDAC1 can be explained by the fact that HDAC1 induces VEGF pathway[36].

Breast

EGFR pathway activation confers sensitivity to a drug targeting ERBB2 (**Supplementary Fig. S3d**). This is a well known fact[37,38] and is unfortunately the only clear top hit for breast tissue.

Lung SCLC

Targeting IGF1R and NTRK1 when activation of TNFa pathway confers sensitivity (**Supplementary Fig. S3j**). Indeed, IGF1R targeted therapies may have a role in the treatment of SCLC in combination with chemotherapy or as maintenance therapy[39]. Treatment of cells expressing NTRK1 fusions with inhibitors of TRKA kinase activity inhibited autophosphorylation of TRKA and cell growth[40,41].

MCL1 showed stronger correlation with adverse prognostic features in SCLC[40], which might explain its sensitivity in presence of MAPK activation in our result (**Supplementary Fig. S3j**).

Pancreas

From our analysis, many targets confer sensitivity when EGFR pathway is activated (**Supplementary Fig. S3m**), especially MEK1/2[42]. In pancreatic ductal adenocarcinoma, EGFR activity is essential in inducing MEK/ERK activity, a requirement for epithelial transformation[43].

SUPPLEMENRARY METHODS

MACAU: Algorithm

General description

Macau trains a Bayesian model for collaborative filtering by also incorporating side information on rows and/or columns to improve the accuracy of the predictions. Drug response matrix (**IC50**) can be predicted using side information from both drugs and cell lines. We use protein target as drug side information and transcriptomics/pathway as cell line side information. Each side information matrix is then transformed into a matrix of N latent dimension by a link matrix. Drug response is then computed by a matrix multiplication of the 2 latent matrices. Macau employs Gibbs sampling to sample both the latent vectors and the link matrix, which connects the side information to the latent vectors. It supports high-dimensional side information (e.g., millions of features) by using conjugate gradient based noise injection sampler.

Probabilistic Matrix Factorization

One of the successful approach to handle matrix factorization for partially observed data is Probabilistic Matrix Factorization (PMF). The main notion behind the PMF is to find a factorization that minimize the root mean square error (RMSE) on the observed data, and maintain good performance on those observed data considered for test set, with the assumption of Gaussian noise in the data. For example, to factorize drug response IC50 using PMF, it represents each row (drugs) and each column (cell lines) by a latent vector of size L and then find the

$$min_{D,C} \ \sum_{(i,j)\in I_{IC50}} \ (IC50_{i,j} - D_i^T C_j) \ + \ \lambda_D \|D\|_F^2 \ + \ \lambda_C \|C\|_F^2 \quad (1)$$

where $IC50_{i,j} \in IC50$ are the observed data, λ_D and λ_C are regularization parameter and greater than zero, and $\| . \|_F$ denotes the Frobenius norm. PMF, Indeed, use a linear model with Gaussian observation noise. Accordingly, last two term in PMF optimization problem (4) are derived from Gaussian prior with zero mean on both latent variables, D_i and C_j, and a Gaussian noise model on $IC50_{i,j}$.

Bayesian PMF for IC50

However, the generalization ability of PMF will decrease when the sparsity of the matrix is increased. To overcome this issue, Bayesian PMF (BPMF) suggests a fully Bayesian treatment of the PMF approach by introducing common multivariate Gaussian priors for the latent variables; one for rows (drugs) and one for columns (cell lines). BPMF also places the Normal-Wishart priors over the row and column hyperparameters:$\Theta_D$andΘ_C.

Θ_D= {μ_D,Λ_D}and Θ_C= {μ_C,Λ_C}are defined as the row and column hyperparameters, where μ_D and Λ_D(μ_C and Λ_C) are the mean and precision matrix of Gaussian prior for drugs (cell lines). The BPMF model is then expressed as

$$p(D, \mu_D, \ \Lambda_D|\Theta_0) = \prod_{i=1} \ N(D_i|\ \mu_D, \Lambda_D^{-1}) NW(\mu_D, \Lambda_D|\Theta_0) \quad (2)$$

$$p(C, \mu_C, \ \Lambda_C|\Theta_0) = \prod_{j=1} \ N(C_j|\ \mu_C, \Lambda_C^{-1}) NW(\mu_C, \Lambda_C|\Theta_0) \quad (3)$$

Where *N* and *NW* denote the normal and Normal-Wishart distributions and Θ_0 are the fixed hyperparameters of the Normal-Wishart hyperprior. Like PMF, BPMF also uses a linear model with Gaussian observation noise.

$$p(IC50|D, C, \alpha_{IC50}) = \prod_{(i,j)\in I_{IC50}} N(IC50_{i,j}|\ D_i^T C_j,\ \alpha_{IC50}^{-1}) \quad (4)$$

where $\alpha_{IC50} > 0$ is the precision parameter. In BPMF, α_{IC50} is assumed to be known.

Proposed model

In order to deliver more accurate IC50 matrix completion, we extend BPMF by incorporating extra information available about drugs and cell lines; which is referred as the side information. This leads to have more accurate factorization. Similarly to BPMF, we suggest IC50 has a Gaussian noise model with precision $\alpha_{IC50} > 0$, as expressed in (2). In order to incorporate the drug and cell line features, we can rewrite the Gaussian prior (2) (3), used in BPFM, for the latent variable $D_i(C_i)$ of a drug *i* (cell line *j*):

$$p(D_i, |\mu_D, \Lambda_D) = N(D_i|\ \mu_D, \Lambda_D^{-1}) \quad (5)$$

$$p(C_j, |\mu_C, \Lambda_C) = N(C_j|\ \mu_C, \Lambda_C^{-1}) \quad (6)$$

where μ_D and Λ_D (μ_C and Λ_C) are the mean and precision matrix of Gaussian prior for drugs (cell lines), respectively. To incorporate the drug's feature (target) $x_i \in R^{F_{drug}}$ (the cell line's features $z_j \in R^{F_{cell}}$), we incorporate a term $\beta_{drug}^T\ x_i$ ($\beta_{cell}^T\ z_j$) into Gaussian mean μ_D (μ_C). Then the equations (5) and (6) are then expressed as

$$p(D_i, |x_i,\ \mu_D, \Lambda_D) = N(D_i|\ \mu_D + \beta_{drug}^T\ x_i, \Lambda_D^{-1}) \quad (7)$$

$$p(C_j, |z_j,\ \mu_C, \Lambda_C) = N(C_j|\ \mu_C + \beta_{cell}^T\ z_j, \Lambda_C^{-1}) \quad (8)$$

Where $\beta_{drug} \in R^{F_{drug} \times L}$ ($\beta_{cell} \in R^{F_{cell} \times L}$) is the weight matrix for the drugs (cell lines) features and F_{drug} (F_{cell}) is the dimensionality of the drugs (cell lines) features. Equations (7) and (8) offers the linear model for latent vectors. For example, for the drugs with no observation in IC50, the distribution of their latent variable are fully determined by (7). In contrast, for the drugs with has many observations in IC50, their features have only a small effect.

We further consider a zero mean multivariate normal as it's prior, to have a full Bayesian treatment for β_{drug}. A zero mean multivariate normal prior is defined in the following way.

$$p(\beta_{drug}, |\ \Lambda_D, \lambda_{\beta_{drug}}) = N(vec(\beta_{drug})|\ 0,\ \Lambda_D^{-1}\ \ (\lambda_{\beta_{drug}} I)^{-1}) \quad (9)$$

$$\propto \lambda_{\beta_{drug}}^{\frac{F_{drug}L}{2}}\ |\Lambda_D|^{\frac{L}{2}}\ exp(\tfrac{1}{2}\ \lambda_{\beta_{drug}}\ tr(\beta_{drug}\Lambda_D^{-1}\beta_{drug}^T) \quad (10)$$

where $\otimes$ denotes the Kronecker product, and $vec(\beta_{drug})$ denotes the vectorization of β_{drug} .

$\lambda_{\beta_{drug}} \geq 0$ is the diagonal element of the precision matrix, and Λ_D is the precision matrix of the latent variable for genes, that has a key role in the development of an efficient computational noise injection sampler discussed later.

We also place a gamma distribution hyperprior on $\lambda_{\beta_{drug}}$, due to the fact that the choice of $\lambda_{\beta_{drug}}$ is problem dependent.

$$p(\lambda_{\beta_{drug}}|\mu,\ \nu) = \ gamma(\lambda_{\beta_{drug}}|\ \mu,\ \nu)\ \propto\ \lambda_{\beta_{drug}}^{\frac{\nu}{2}-1} exp(-\frac{\nu}{2\mu}\lambda_{\beta_{drug}}) \qquad (11)$$

where μ and ν are fixed hyperparameters, which are both set to 1 in the experiments. The same full Bayesian treatment is developed for β_{cell}.

Gibbs Sampling

Gibbs sampling is the simplest and most established Markov chain Monte Carlo (MCMC), which allows to sample from the exact posterior of the model variables. Gibbs sampler iteratively samples each model variable from its conditional distribution while keeping others fixed.

From (2) and (3), it is straightforward to derive block Gibbs sampler for each latent vector D_i and C_i , and for the parameters of the Gaussian priors $\mu_D, \mu_C, \Lambda_D\ , \Lambda_C$. This allows us to generate multiple approximations of D_i and C_i latent matrices and then get a better approximation out of it. So, in this section we present the conditional distributions of the Gibbs sampler for all variables except for $\beta_{drug}$and β_{cell}. The outline of Gibbs sampling for our proposed model is as follows: Based on (5) and (6), the conditional probability for latent vectors D_i and C_j are:

$$p(D_i|\ IC50, C, \Theta_D, x, \beta, \lambda, \alpha, \Lambda_D) = \ N(D_i|\ \mu_{D_i}^*, \qquad [\Lambda_{D_i}^*]^{-1}) \qquad (12)$$

$$\propto \prod_{j\in\ I_{IC50(i,)}} N(IC50_{i,j}|\ D_i^T C_j\ ,\ \alpha^{-1}) \qquad (13)$$

$$\times\ N(D_i|\ \mu_D +\ \beta_{drug}^T\ x_i, \Lambda_D^{-1})$$

where

$$\Lambda_{D_i}^* = \Lambda_D + \alpha \sum_{(i,j)\in\ I_{IC50(i,)}} C_j C_j^T$$

$$\mu_{D_i}^* = [\Lambda_{D_i}^*]^{-1}(\Lambda_D(\mu_D +\ \beta_{drug}^T\ x_i) +\ \alpha \sum_{(i,j)\in\ I_{IC50(i,)}} C_j\ IC50_{i,j}\ )$$

From the above equations, we see that if drug *i* does not have features then $\beta_{drug}^T\ x_i$ is 0. We also place the Normal-Wishart hyperprior for μ_D and Λ_D, same as being used in BPMF.

$$p(\mu_D,\ \Lambda_D|\Theta_0) = \ N(\mu_D|\ \mu_0^*,\ (\beta_0^*\Lambda_D)^{-1})NW(\Lambda_D|W_0,\ \nu_0) \qquad (14)$$

Where NW denote Normal Wishart distributions. In this study, we set $\mu_0 = 0$, $\beta_0 = 2$ and $\nu_0 = L$ and $,W_0$ to the identity matrix for both drug and cell line. Combining the hyperprior (13) with (4) we get the following conditional probability

$$p(\mu_D,\ \Lambda_D|D, x, \beta_{drug}, \Theta_0) = \ N(\mu_D|\ \mu_0^*,\ (\beta_0^*\Lambda_D)^{-1})NW(\Lambda_D|W_0^*,\ \nu_0^*) \qquad (15)$$

Where

$$\mu_0^* = \frac{\beta_0\mu_0 + N_D\bar{D}}{\beta_0 + N_D} \qquad \beta_0^* = \beta_0 +\ N_D \qquad \nu_0^* = \nu_0 + N_D,$$

$$[W_0^*]^{-1} = W_0^{-1} + N_D\underline{S} + \frac{\beta_0 N_D}{\beta_0 + N_D}(\mu_0 + \underline{D})(\mu_0 + \underline{D})^T$$

$$\underline{U} = \frac{1}{N_D}\sum_{i=1}^{N_D}(D_i - \beta_{drug}^T x_i) \qquad \underline{S} = \frac{1}{N_D}\sum_{i=1}^{N_D}(D_i - \beta_{drug}^T x_i)(D_i - \beta_{drug}^T x_i)^T$$

While in BPMF the Gaussian priors model the latent variables D_i, in our proposed work the Gaussian priors model the residual $D_i - \beta_{drug}^T x_i$ instead; this being the key difference in comparison with BPMF.

The conditional probability for precision parameter if the weight vector, $\lambda_{\beta_{drug}}$, can be obtained from (9) and (11)

$$p(\lambda_{\beta_{drug}} | \beta_{drug}, \Lambda_D, \mu, \nu) = gamma(\lambda_{\beta_{drug}} | \hat{\mu}, \hat{\nu}) \qquad (16)$$

Where

$$\hat{\nu} = F_{drug}L + \nu \qquad \hat{\mu} = \frac{(F_{drug}L + \nu)\mu}{\nu + \mu\, tr(\beta_{drug}\Lambda_D^{-1}\beta_{drug}^T)}$$

Note that the conditional probability for $\lambda_{\beta_{cell}}$ has exactly the same form.

Noise Injection Sampler

Based on Equations (8) and (9), the conditional probability for β_{drug} can be reformulated as

$$p\left(\beta_{drug} \middle| \mu_{drug}, \Lambda_D, D, x, \lambda_{\beta_{drug}}\right)$$
$$\propto exp\,(-\frac{1}{2}\sum_{i=1}^{N_D}(D_i - \mu_D - \beta_{drug}^T x_i)^T \Lambda_D (D_i - \mu_D - \beta_D^T x_i)$$
$$-\frac{1}{2}\lambda_{\beta_{drug}}\, tr(\beta_{drug}\Lambda_D^{-1}\beta_{drug}^T)) \qquad (17)$$

Let X represents $X = [x_1, \ldots, x_N]$ and D denotes U= $[D_1 - \mu_D, \ldots, D_N - \mu_D]$. As both the likelihood and prior terms contain Λ_D, thus it can be factored out:

$$p(\beta_{drug} | \mu_{drug}, \Lambda_D, D, x, \lambda_{\beta_{drug}})$$
$$\propto exp(-\tfrac{1}{2}tr[((D - X\beta_{drug})^T(D - X\beta_{drug}) + \lambda_{\beta_D}(\beta_{drug}\beta_{drug}^T)\Lambda_D]) \qquad (18)$$

Then, the parameters of the Gaussian (mean and precision) can be reformulated as:

$$p(\beta_{drug} | \mu_{drug}, \Lambda_D, D, x, \lambda_{\beta_{drug}})$$
$$\propto exp(-\tfrac{1}{2}\, vec\,(\beta_{drug} - \hat{\beta}_{drug})^T(\Lambda_D \; (X^T X + \lambda_{\beta_{drug}} I))\, vec\,(\beta_{drug} - \hat{\beta}_{drug})) \qquad (19)$$

where $\hat{\beta}_{drug} = (X^T X + \lambda_{\beta_{drug}} I) X^T D$ is the mean and Λ_D $(X^T X + \lambda_{\beta_{drug}} I)$ is the precision of the posterior.

However, the typical methods to sample from multivariate Gaussian distribution, quickly becomes expensive and demanding as the feature-dimension becomes large. For example, for a dimension of 10,000 for F_{drug} , the size of the precision matrix is ($F_{drug} \times F_{drug}$) 10^8, which is computationally intensive. Alternatively, drawing a sample from (19) for β_{drug} can be made by solving a linear system for $\hat{\beta}_{drug}$:

$$(X^T X + \lambda_{\beta_{drug}} I) \hat{\beta} = X^T (U + E_1) + \sqrt{\lambda_{\beta_{drug}}} E_2 \quad (20)$$

where each row of matrices $E_1 \in R^{N_{drug} \times L}$ and $E_2 \in R^{F_{drug} \times L}$ is sampled from $N(0, \Lambda_D^{-1})$. When $X$is sparse, we can speed up the process of solving this linear system by using interactive method such as conjugate gradient (CG).

Note that the conditional probability for β_{cell} has exactly the same form.

DRUG TARGET PREDICTION

Preprocessing

All descriptors excluding Morgan Fingerprints were standardized by mean centering and scaling to unit variance.

Descriptors

Using RDKit, the following physicochemical descriptors were calculated: Molecular Weight, n H-bond acceptors, n H-bond donors, n Rotatable bonds, LogP, and FPSA.
The molecular structure was described using Morgan Fingerprints generated with RDKit, with a radius of 3, and a length of 1024 bits.
In addition to chemical descriptors, protein information was incorporated by calculating physicochemical properties for every protein as was done previously [1]. The resulting protein descriptor matrix contains 510 descriptors for 3644 proteins.

Datasets

Training set data and protein information were gathered from the ChEMBL database v22.1 as was done previously.[1,2] In addition, records from functional or ADMET assays were excluded, leaving only binding assays. In the case of duplicate records, the median pChEMBL activity value was chosen. In total, the dataset contained 426,043 unique ligand-protein activity records, targeting a total of 3644 proteins. (train_full_c9.txt) Prediction was performed on targets with at least 50 records in the training, totalling 1035 targets.

Machine Learning

Fitting and predicting was done by a regression Deep Neural Network model constructed using Keras and Theano with the parameters described in Table I.

Table I: Model parameters.

n_layers/ n_neurons	2000, 100	Number of neurons in each hidden layer
p_dropout	0.5, 0.5	Dropout probability per layer, only on hidden layers
validation_split	0.2	Fraction of training data used as validation data to monitor loss for early stopping
max_epochs	2000	Maximum number of epochs to train
patience	20	Maximum number of epochs without improvement before early stopping
batch_size	128	Number of samples per mini-batch
optimizer	SGD	Stochastic Gradient Descent with Nesterov Momentum and linear learning rate decay based on max epochs
l	0.001	Learning rate
mom	0.9	Nesterov momentum
loss	MSE	Loss function

activation	ReLU	Hidden layer neuron activation function
output_activation	ReLU	Output neuron activation function

References

1. Lenselink, Eelke B. et al. “Beyond the Hype: Deep Neural Networks Outperform Established Methods Using a ChEMBL Bioactivity Benchmark Set.” Journal of Cheminformatics 9 (2017): 45. *PMC*. Web. 22 Jan. 2018.
2. Gaulton, A., et al., ChEMBL: a large-scale bioactivity database for drug discovery. Nucleic Acids Research, 2012. 40(D1): p. D1100-D1107.

Supplementary tables

	**Setting 1**<br>predicting new cell lines	**Setting 2**<br>predicting new drugs	**Setting 3**<br>predicting existing drugs<br>on existing cell lines	**Setting 4**<br>predicting new drugs on new cell lines
**use case**	- Personalized medicine	- Drug repositioning	- prioritization for new experiments<br>- **Interaction matrix generation**	- Personalized medicine with previously untested drugs<br>- **Quality control of the interaction matrix**
**drug features**	optional	**required**	optional	**required**
**cell line features**	**required**	optional	optional	**required**
**cross validation**	10 fold CV	10 fold CV	NA	2 x 10 fold CV
**prediction metrics**	For each drug, pearson correlation of observed versus predicted IC50 across all cell lines.	For each cell line, pearson correlation of observed versus predicted IC50 across all drugs.	Pearson correlation of observed versus predicted IC50 for all drug-cell line pairs.	Pearson correlation of observed versus predicted IC50 for all drug-cell line pairs.

Supplementary Table S1: Different settings for drug response prediction

	Ridge-LASSO	Random Forest	XGBOOST
**Macau**	**S1**: 0.30 vs 0.30 (p=0.92)<br>**S2**: 0.42 vs 0.12 (**p< 2.2e-16**)<br>**S4**: 0.44 vs 0.41 (p=0.24)	**S1**: 0.30 vs 0.31 (p=0.47)<br>**S2**: 0.42 vs 0.38 (**p< 2.2e-16**)<br>**S4**: 0.44 vs 0.42 (p=0.34)	**S1**: 0.30 vs 0.31 (p=0.21)<br>**S2**: 0.42 vs 0.19 (**p< 2.2e-16**)<br>**S4**: 0.44 vs 0.39 (**p=0.0067**)
**Ridge-LASSO**		**S1**: 0.30 vs 0.31 (p=0.42)<br>**S2**: 0.12 vs 0.38 (**p< 2.2e-16**)<br>**S4**: 0.41 vs 0.42 (p=0.61)	**S1**: 0.30 vs 0.31 (p=0.17)<br>**S2**: 0.12 vs 0.19 (**p< 2.2e-16**)<br>**S4**: 0.41 vs 0.39 (p=0.52)
**Random Forest**			**S1**: 0.31 vs 0.31 (p=0.60)<br>**S2**: 0.38 vs 0.19 (**p< 2.2e-16**)<br>**S4**: 0.42 vs 0.39 (p=0.12)

Supplementary Table S2: Performance comparison of different algorithms (Macau, ridge/LASSO, Random Forest, XGBOOST) in different settings (1, 2 and 4), with PROGENy and/or drug target as input features.

response	repetition	test set	latent	N samples	Pearson correlation	RMSE
IC50	10	10%	10	600	0.932 (sd=0.0011)	0.966 (sd=0.015)
IC50	10	20%	10	600	0.931 (sd=0.00053)	0.982 (sd=0.007)
IC50	10	30%	10	600	0.929 (sd=0.00062)	0.996 (sd=0.005)
IC50	10	50%	10	600	0.927 (sd=0.00036)	1.038 (sd=0.005)
IC50	10	70%	10	600	0.919 (sd=0.0002)	1.135 (sd=0.003)
IC50	10	99%	10	600	0.834 (sd=0.004)	2.239 (sd=0.044)

Supplementary Table S3: Prediction performance for missing value imputation

	Target-GEX	Target-PROGENy11	Predicted target -PROGENy11	Target-SNP_CNV
**aero_dig_tract**	0.456 (sd=0.168)	0.441 (sd=0.189)	0.331 (sd=0.214)	0.418 (sd=0.174)
**bone**	0.412 (sd=0.18)	0.401 (sd=0.177)	0.325 (sd=0.194)	0.376 (sd=0.168)
**brain**	0.433 (sd=0.175)	0.426 (sd=0.166)	0.347 (sd=0.178)	0.394 (sd=0.171)
**breast**	0.426 (sd=0.171)	0.407 (sd=0.17)	0.327 (sd=0.2)	0.372 (sd=0.176)
**colon**	0.433 (sd=0.172)	0.395 (sd=0.164)	0.347 (sd=0.183)	0.396 (sd=0.172)
**kidney**	0.38 (sd=0.189)	0.392 (sd=0.191)	0.281 (sd=0.202)	0.36 (sd=0.184)
**leukemia**	0.393 (sd=0.148)	0.388 (sd=0.155)	0.299 (sd=0.19)	0.35 (sd=0.156)
**liver**	0.388 (sd=0.202)	0.334 (sd=0.223)	0.271 (sd=0.207)	0.323 (sd=0.216)
**lung_NSCLC**	0.421 (sd=0.171)	0.406 (sd=0.181)	0.302 (sd=0.184)	0.351 (sd=0.163)
**lung_SCLC**	0.419 (sd=0.15)	0.415 (sd=0.153)	0.333 (sd=0.171)	0.381 (sd=0.176)
**lymphoma**	0.417 (sd=0.164)	0.408 (sd=0.153)	0.353 (sd=0.167)	0.332 (sd=0.168)
**ovary**	0.441 (sd=0.175)	0.418 (sd=0.178)	0.325 (sd=0.201)	0.385 (sd=0.189)
**pancreas**	0.367 (sd=0.217)	0.337 (sd=0.213)	0.268 (sd=0.242)	0.379 (sd=0.207)
**skin**	0.448 (sd=0.177)	0.454 (sd=0.158)	0.347 (sd=0.187)	0.409 (sd=0.169)
**soft_tissue**	0.402(sd=0.194)	0.359 (sd=0.207)	0.315 (sd=0.202)	0.361 (sd=0.219)
**stomach**	0.437(sd=0.181)	0.376 (sd=0.199)	0.263 (sd=0.211)	0.383 (sd=0.187)

Supplementary Table S4: Tissue specific prediction performance for setting 4: prediction of new drugs on new cell lines on GDSC dataset.

	target_GEX	target_PROGENy11
**aero_dig_tract**	0.371 (sd=0.132)	0.338 (sd=0.135)
**blood**	0.284 (sd=0.107)	0.287 (sd=0.123)
**breast**	0.239 (sd=0.144)	0.104 (sd=0.127)
**colon**	0.268 (sd=0.132)	0.251 (sd=0.129)
**endometrium**	0.281 (sd=0.14)	0.205 (sd=0.157)
**head**	0.175 (sd=0.132)	0.098 (sd=0.129)
**liver**	0.237 (sd=0.128)	0.159 (sd=0.143)
**lung**	0.127 (sd=0.141)	0.125 (sd=0.146)
**oesophagus**	0.334 (sd=0.143)	0.282 (sd=0.146)
**ovary**	0.102 (sd=0.226)	-0.014 (sd=0.262)
**pancreas**	0.236 (sd=0.218)	0.027 (sd=0.212)
**skin**	0.183 (sd=0.143)	0.075 (sd=0.153)
**stomach**	0.096 (sd=0.23)	0.154 (sd=0.224)
**urinary_tract**	0.3 (sd=0.135)	0.243 (sd=0.141)

Supplementary Table S5: Tissue specific prediction performance for setting 4: prediction of new drugs on new cell lines on CTRPv2 dataset.

pathway	target	Max tissue	Min tissue	Max value	Min value	Max - Min	absolute mean
VEGF	Microtubule stabiliser	colon	Soft tissue	0.351	-0.474	0.825	0.123
Hypoxia	ALK	lymphoma	liver	0.519	-0.369	0.888	0.15
TNFa	HDAC1	bone	pancreas	0.592	-0.427	1.02	0.165
TNFa	PI3Kbeta	brain	skin	0.414	-0.39	0.804	0.024
NFkB	ERBB2	breast	stomach	0.632	-0.637	1.27	0.005
EGFR	dsDNA break	colon	breast	0.478	-0.667	1.15	0.189
EGFR	MEK2	pancreas	bone	0.424	-0.426	0.85	0.002

Supplementary Table S6: Top antagonistic pathway - target pairs across tissues

	correlation	p-value	Adjusted p-value
**aero_dig_tract**	0.15200	4.49e-01	5.85e-01
**bone**	0.31100	4.58e-07	3.66e-06
**brain**	0.18500	1.02e-03	4.53e-03
**breast**	0.36300	1.57e-10	2.51e-09
**colon**	0.13800	6.10e-02	9.76e-02
**kidney**	0.00968	9.11e-01	9.11e-01
**leukemia**	0.00712	9.07e-01	9.11e-01
**liver**	0.05400	6.17e-01	7.05e-01
**lung_NSCLC**	0.16900	1.55e-02	3.09e-02
**lung_SCLC**	-0.10900	3.71e-01	5.40e-01
**lymphoma**	0.20400	2.31e-03	7.38e-03
**ovary**	0.19300	4.11e-02	7.31e-02
**pancreas**	0.26600	9.50e-03	2.17e-02
**skin**	0.24800	1.13e-03	4.53e-03
**soft_tissue**	0.08940	4.75e-01	5.85e-01
**stomach**	0.26900	8.28e-03	2.17e-02

Supplementary Table S7: Correlation between absolute interaction weights and pubmed count.

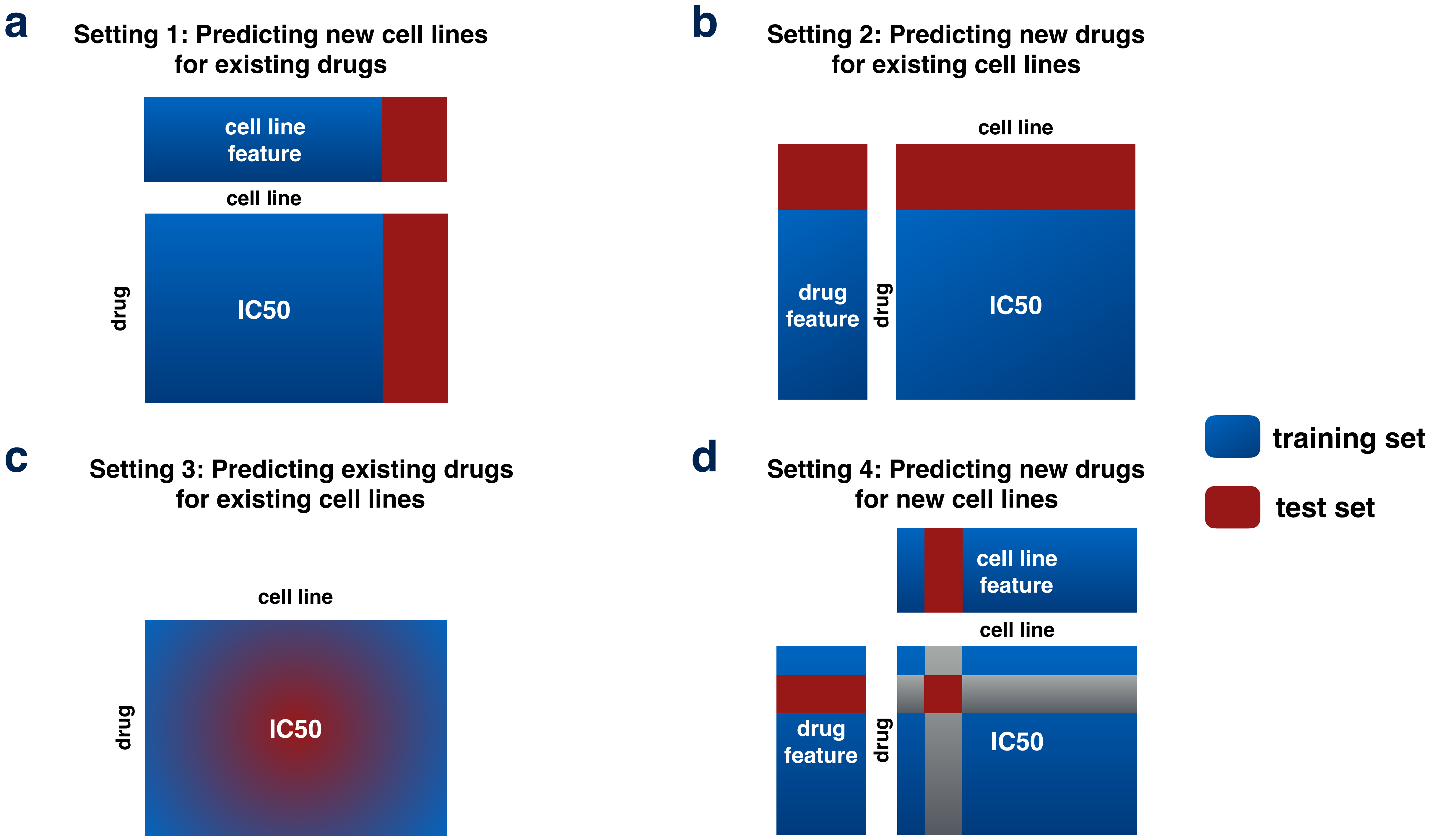

Supplementary Fig. S1. Different settings in drug response prediction. **(a)** Predicting new cell lines for existing drugs. For each drug, we compute the pearson correlation of observed versus predicted IC50 across all cell lines of the test set. **(b)** Predicting new drugs for existing cell lines. For each cell line we compute the pearson correlation of observed versus predicted IC50 across all drugs of the test set. **(c)** Predicting existing drugs for existing cell lines. This is a missing value imputation setting where side information of drug and cell lines are not required, but can be used to improve the result. The test data is defined by a percentage of the whole dataset. We compute the pearson correlation of observed versus predicted IC50 for all randomly chosen drug - cell line pairs of the test set. **(d)** Predicting new drugs for new cell lines. We do 2 simultaneous cross validation on both drug and cell line sides. The test data is defined by association of the test set of the drug side with the test set of the cell lines side. We compute the pearson correlation of observed versus predicted IC50 for all drug - cell line pairs of the test set.

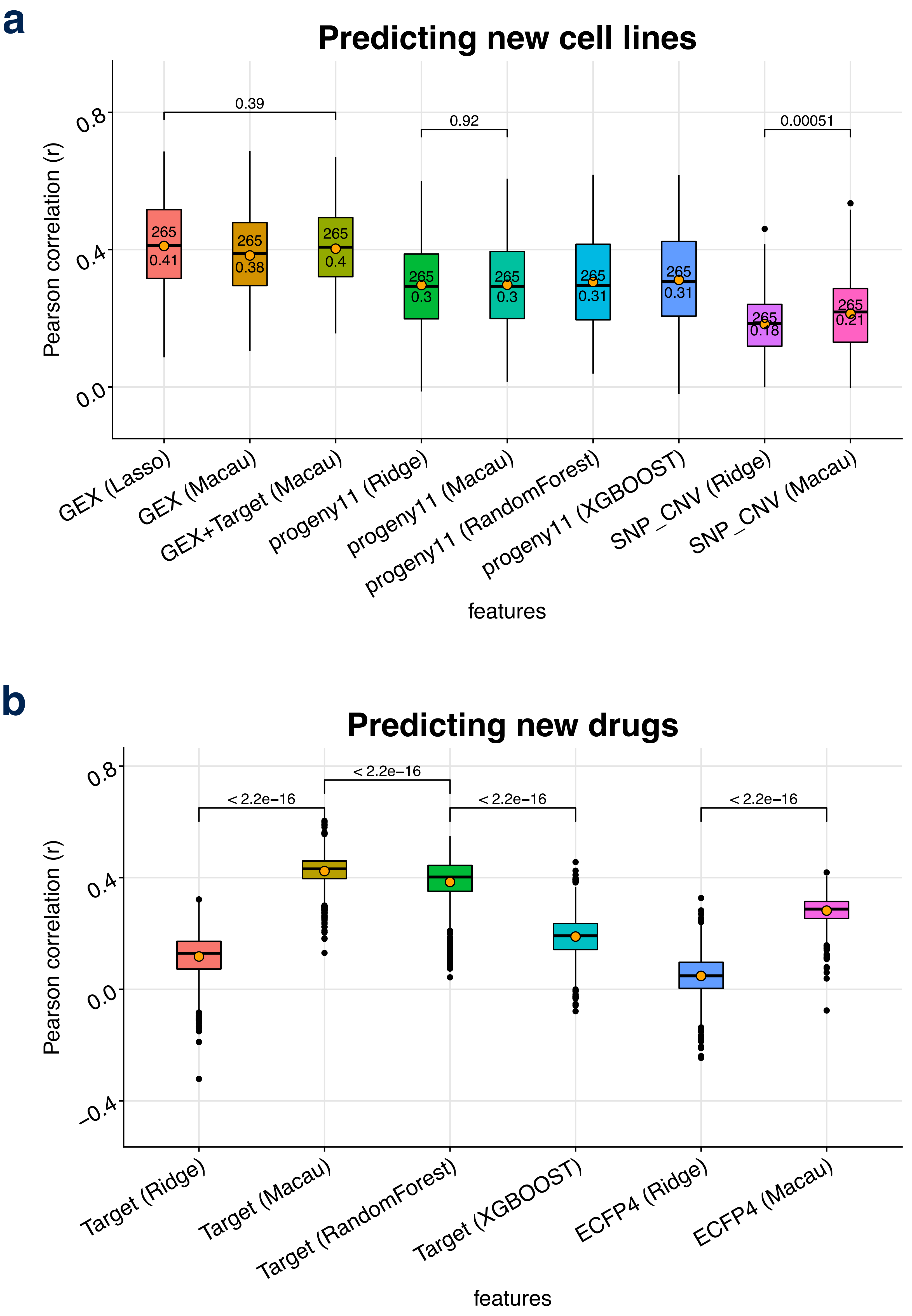

Supplementary Fig. S2. Drug response prediction performance. (a) We compare prediction performance (correlation of observed versus predicted IC50) of existing drugs on new cell lines. We use Macau, ridge/lasso regression, Random Forest and XGBOOST. The features are gene expression, pathway activity, mutation (SNP) and copy number variation (CNV). **(b)** We compare prediction performance of existing cell lines on new drugs. The features are drug protein targets and ECFP4 fingerprint.

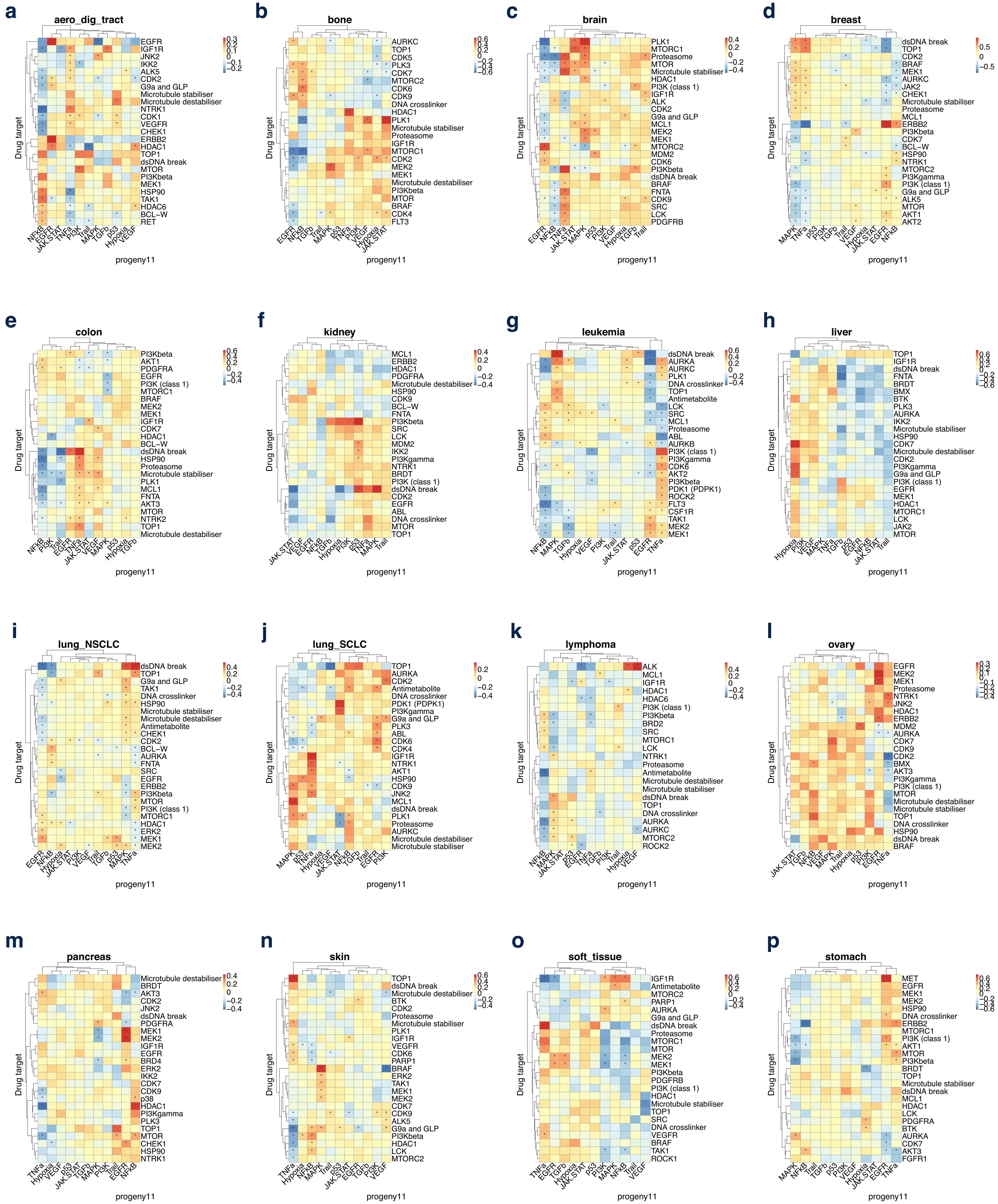

Supplementary Fig. S3. Tissue specific analysis of interaction matrix. We chose 16 tissues in the GDSC panel with at least 20 samples. We kept the targets which have an interaction for at least 1 pathway in the top 5% absolute value. We subset a second time by keeping the top 25 targets with the highest variance across the pathways in term of interaction value.

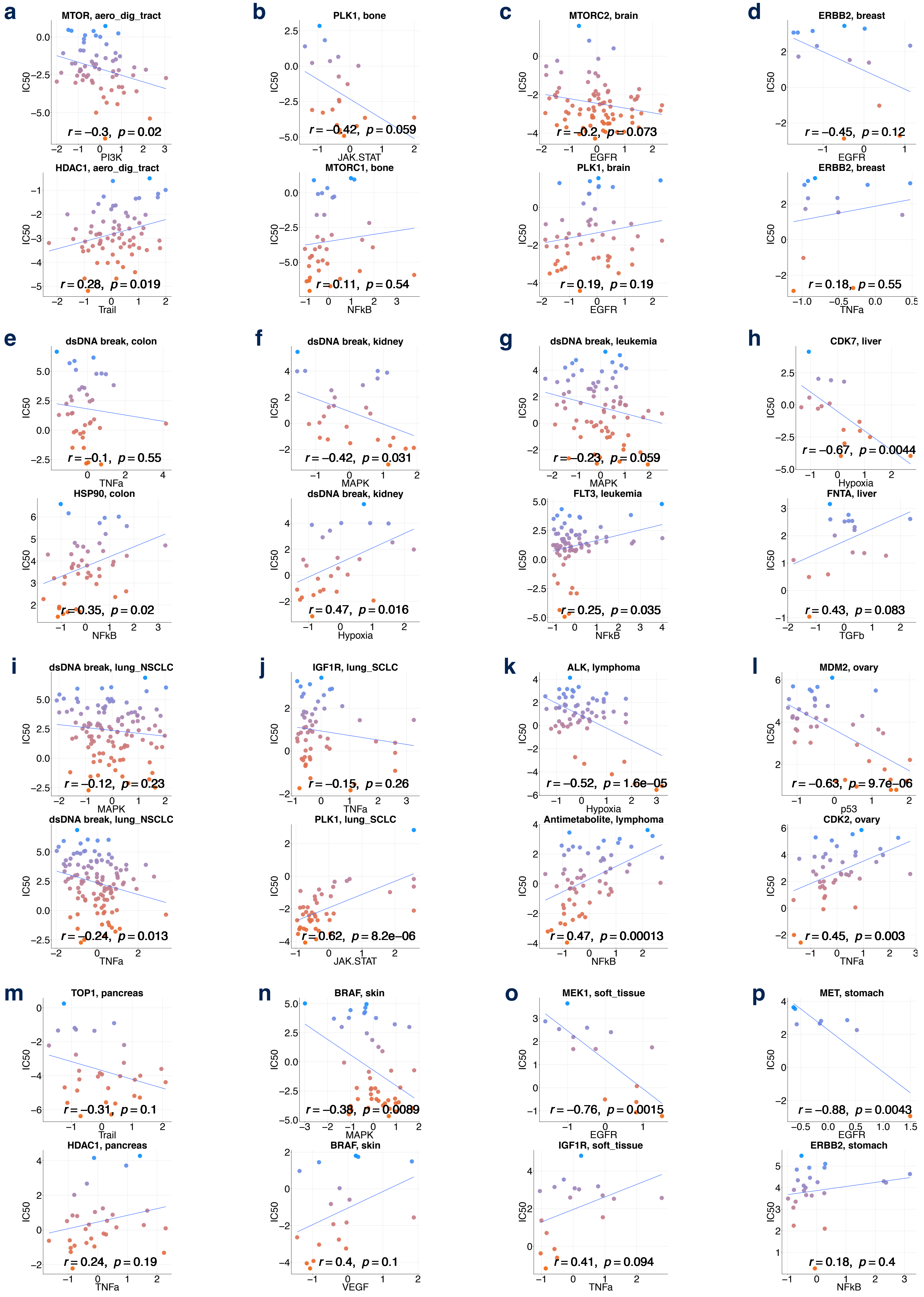

Supplementary Fig. S4. PROGENy as biomarker. For each tissue specific interaction matrix, we select a top positive interaction and a top negative interaction. For both target - pathway pairs, we then find a drug which targets this protein (as described in the manually curated list) and plot its IC50 (log scale) against the corresponding pathway's activity in the specific tissue.

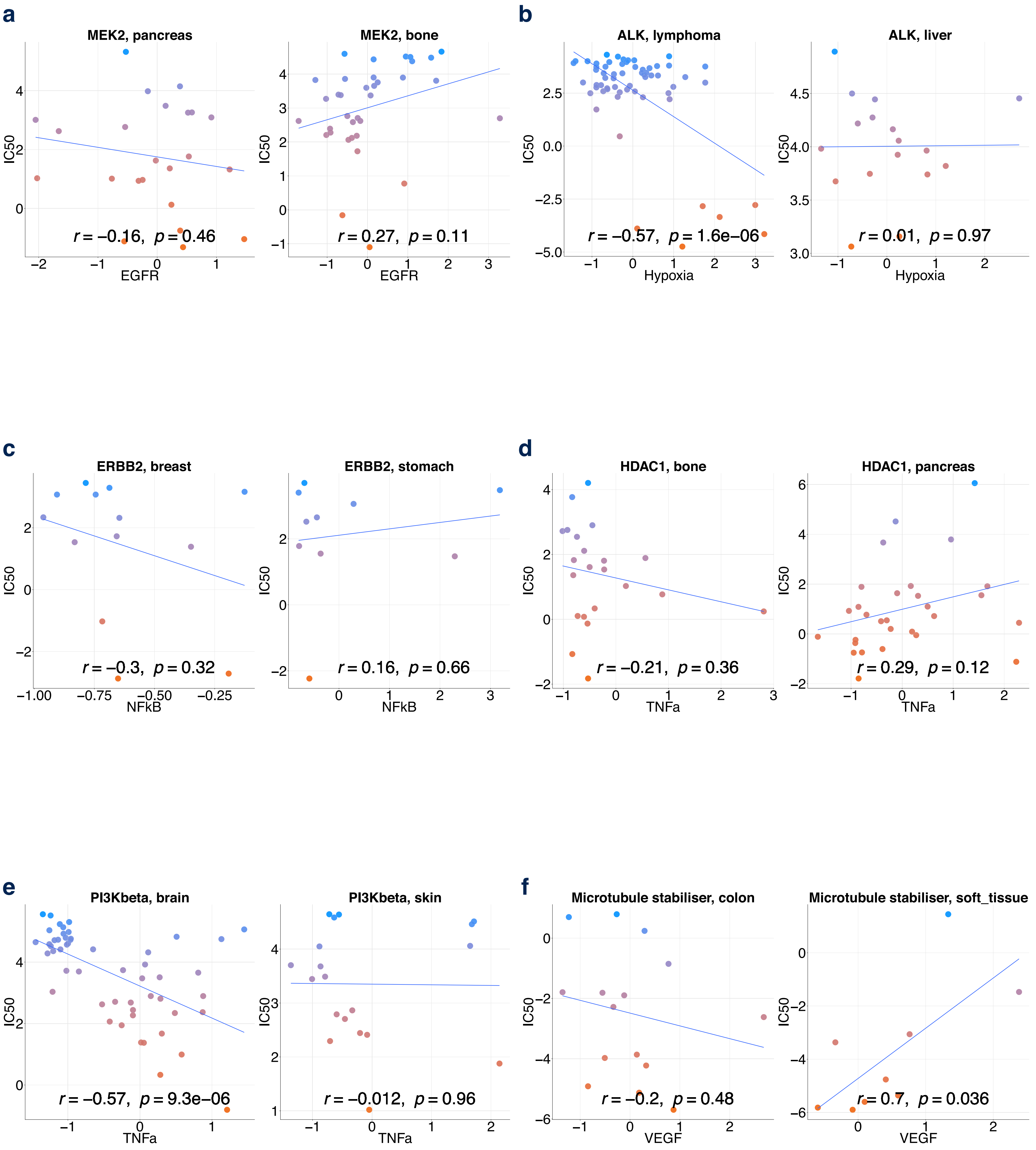

Supplementary Fig. S5. Antagonistic tissues based on target pathway interaction. For all target - pathway pairs which have opposite effect from one tissue to another, we select a drug which specifically targets the protein and plot the drug's IC50 as function of PROGENy activity for the corresponding tissues.

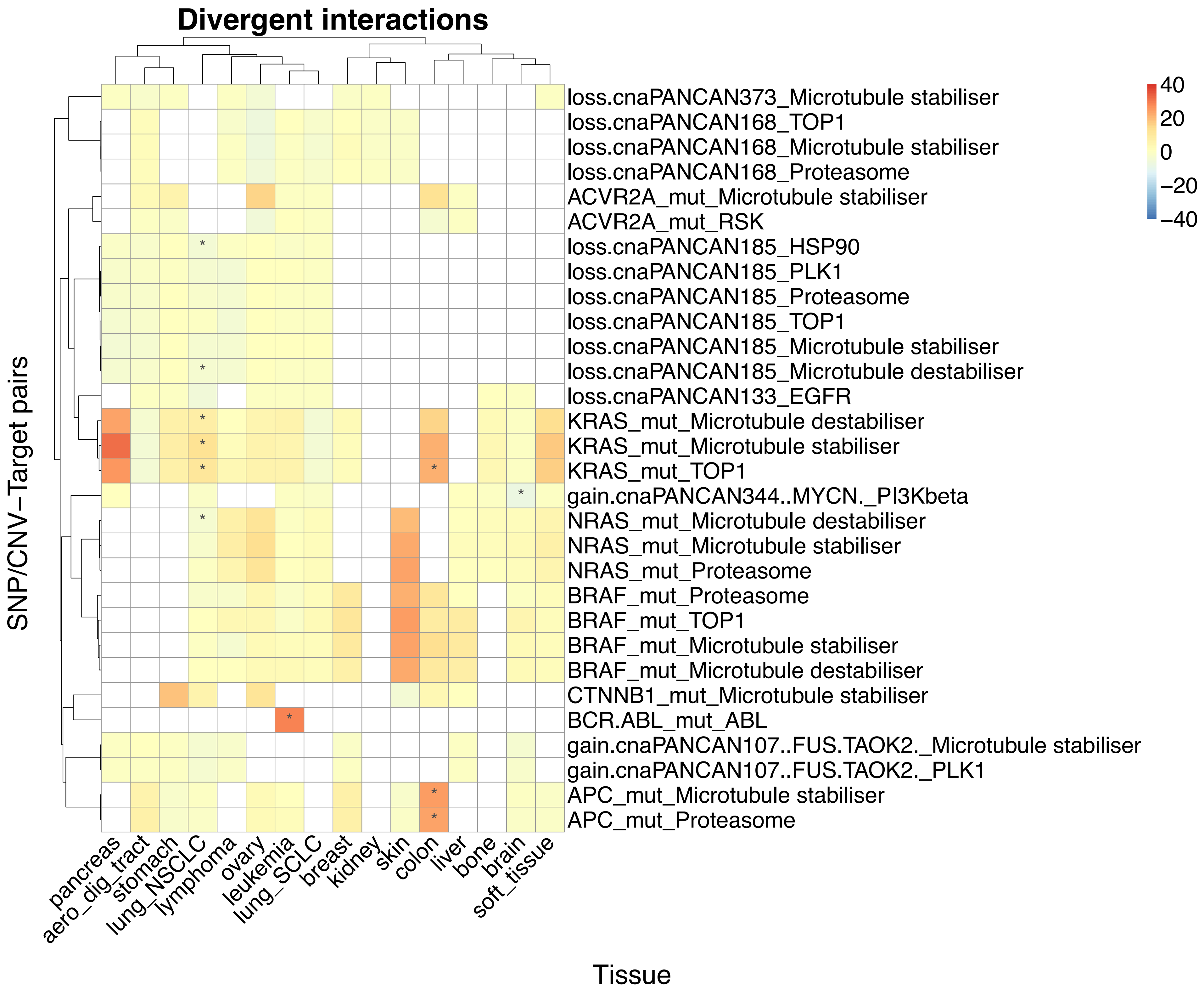

Supplementary Fig. S6. **Feature interaction analysis across tissues for SNP/CNV**. We vectorize all cancer specific interaction matrices between target and SNP/CNV and obtain a matrix of dimension (number of tissues x number of SNP/CNV-target pairs). We do a first subsetting by taking the pairs for which at least one pathway appears in the top 1% highest value, and chose 15 SNP/CNV-target pairs with highest variance of interaction across tissues. We then subset by taking the pairs for which at least one pathway appears in the top 1% lowest value, and chose 15 SNP/CNV-target pairs with highest variance of interaction across tissues. We combine the top hits and then keep the 30 pathway-target pairs. White color indicates when the mutation or CNV is not present.

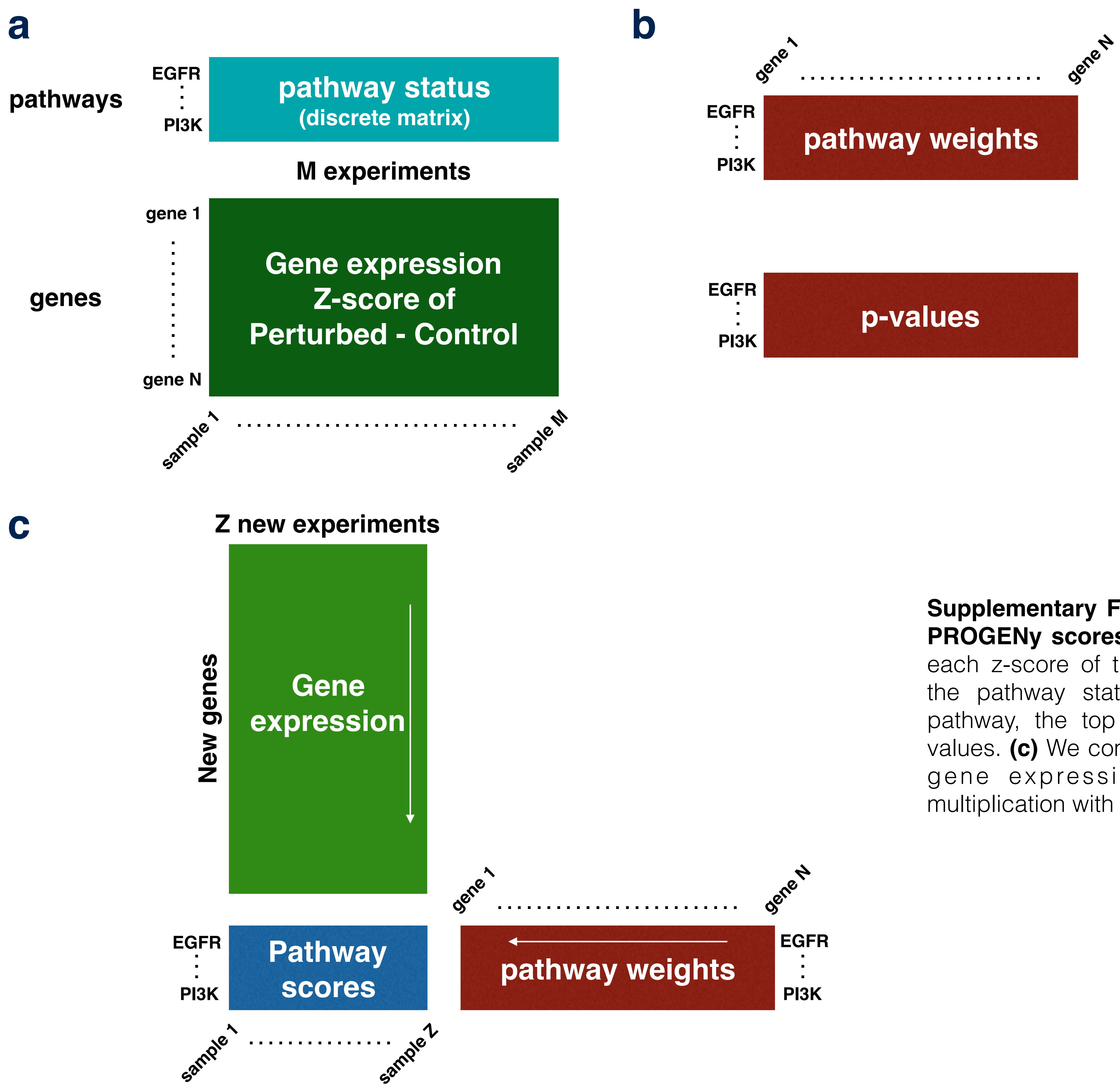

Supplementary Fig. S7. Workflow to produce PROGENy scores. (a) We fit a linear model for each z-score of the perturbation in function of the pathway status. **(b)** We select for each pathway, the top 100 genes with smallest p-values. **(c)** We compute pathway scores for new gene expression dataset by a matrix multiplication with the weight matrix.

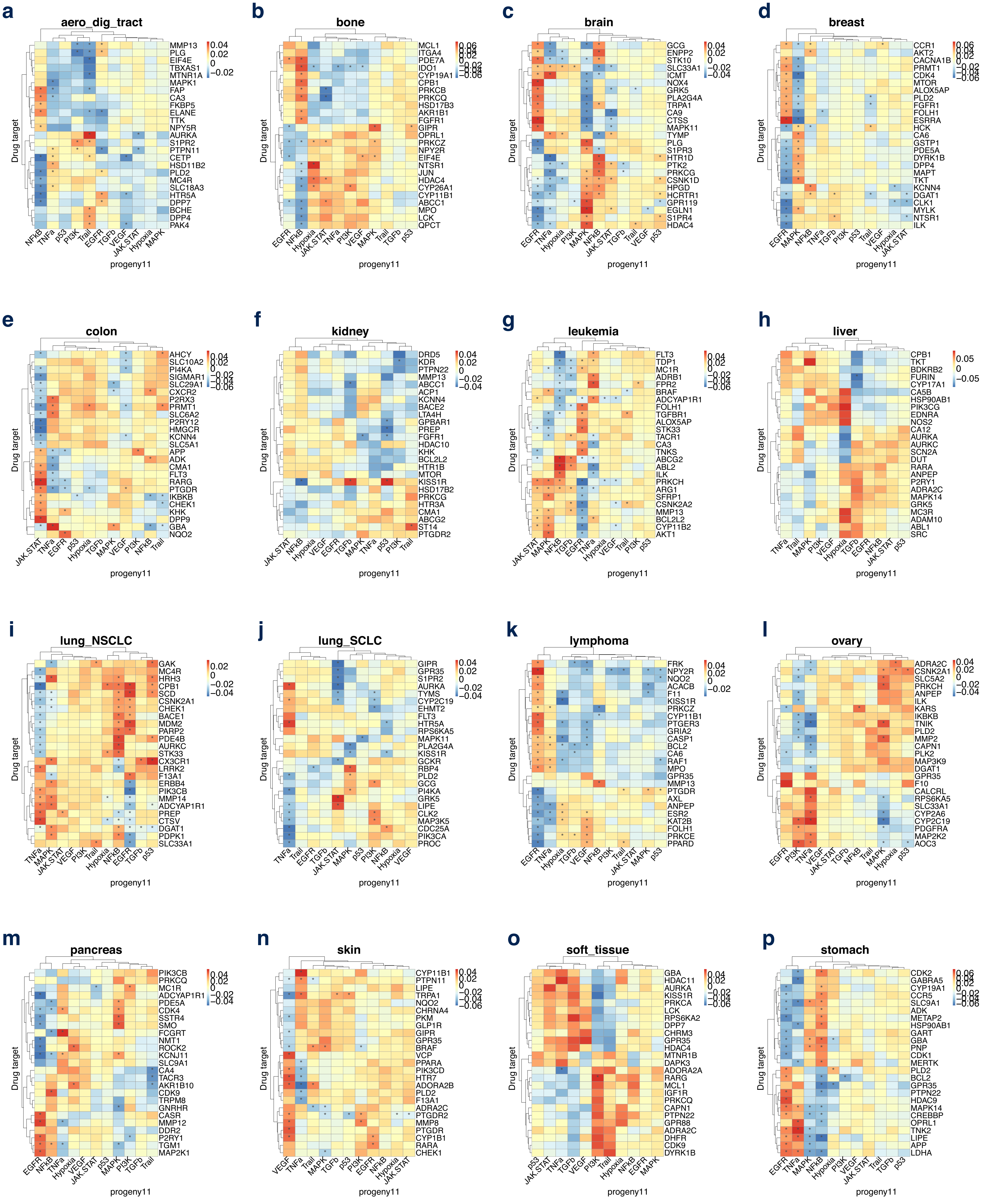

Supplementary Fig. S8. Tissue specific analysis of interaction matrix using predicted drug target. We chose 16 tissues in the GDSC panel with at least 20 samples. We kept the targets which have an interaction for at least 1 pathway in the top 5% absolute value. We subset a second time by keeping the top 25 targets with the highest variance across the pathways in term of interaction value.

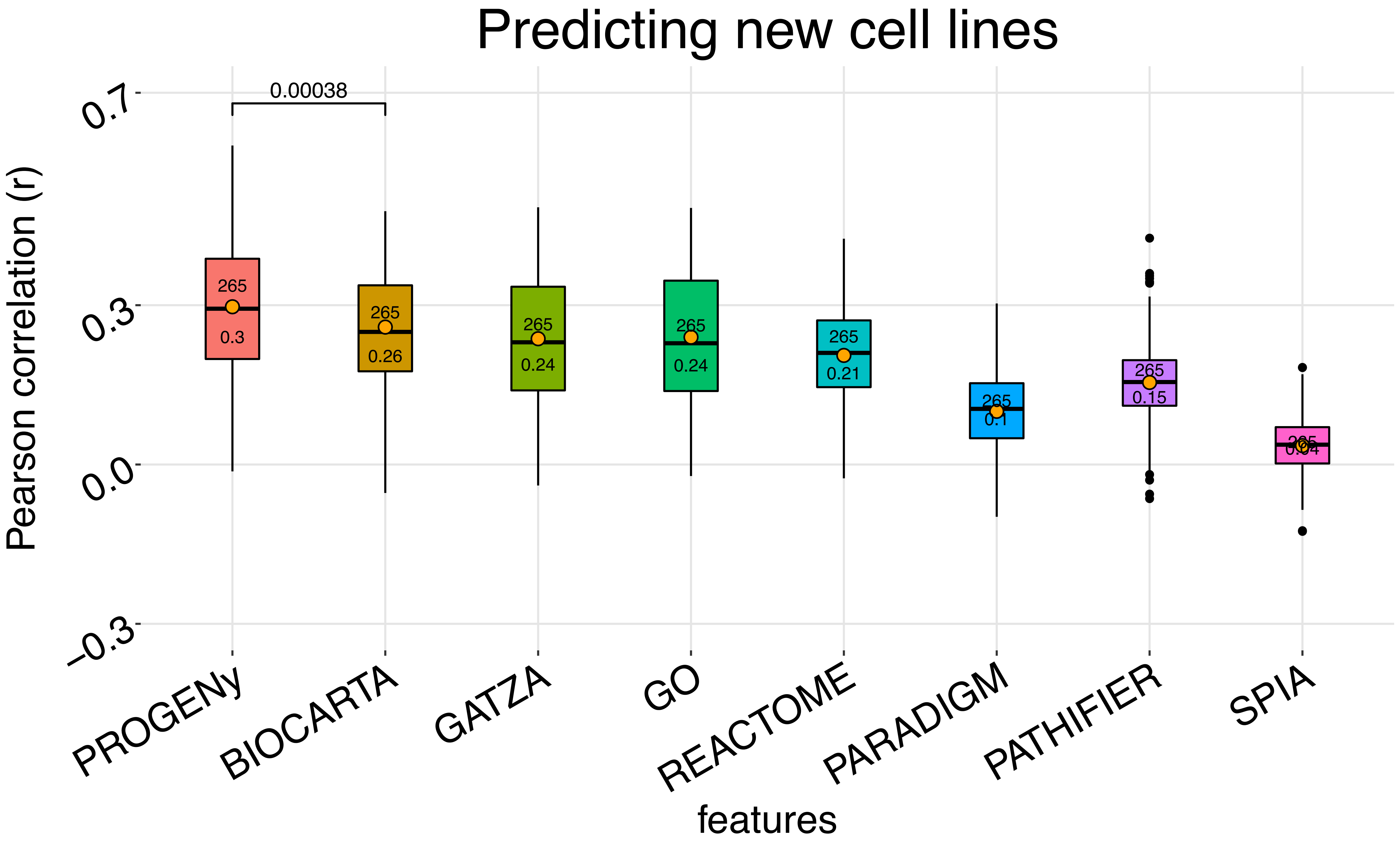

Supplementary Fig. S9. Drug response prediction performance of different pathway methods. We compare prediction performance (correlation of observed versus predicted IC50) of existing drugs on new cell lines. We use elastic net regression. The features are pathway scores derived from different genesets, as described in Schubert et al.
